# Efficacy of underwater endoscopic intermuscular dissection in the management of a rectal neuroendocrine tumor

**DOI:** 10.1055/a-2333-9660

**Published:** 2024-06-18

**Authors:** Suhuan Liao, Silin Huang, Guang Yang, Bo Li, Huizhao Deng, Yi Tan, Guifa He

**Affiliations:** 1 Department of Gastroenterology, South China Hospital, Medical School, Shenzhen University, Shenzhen, China; 2 Department of Nephrology and Rheumatology, Shenzhen Hospital of Integrated Traditional Chinese and Western Medicine, Shenzhen, China; 3 Department of Anesthesiology, South China Hospital, Medical School, Shenzhen University, Shenzhen, China

Endoscopic intermuscular dissection (EID), which is emerging as a therapeutic modality, has garnered recent attention for its efficacy in reducing positive vertical margins, particularly in the management of neuroendocrine tumors (NETs) 1 2 3 . EID procedures are intricate, requiring precise differentiation of the intermuscular space. Herein, we endeavor to employ underwater EID (U-EID) techniques to enhance procedural efficacy.

A 66-year-old woman presented with a subepithelial lesion in the rectum, measuring approximately 7 mm in diameter with a yellowish appearance ( Fig. 1 **a** ). Endoscopic ultrasound indicated the lesion’s location in the deep mucosal and submucosal layers, adjacent to the muscularis propria ( Fig. 1 **b** ). Consequently, U-EID was chosen as the preferred therapeutic approach ( Fig. 2 , Video 1 ).

**Fig. 1:**
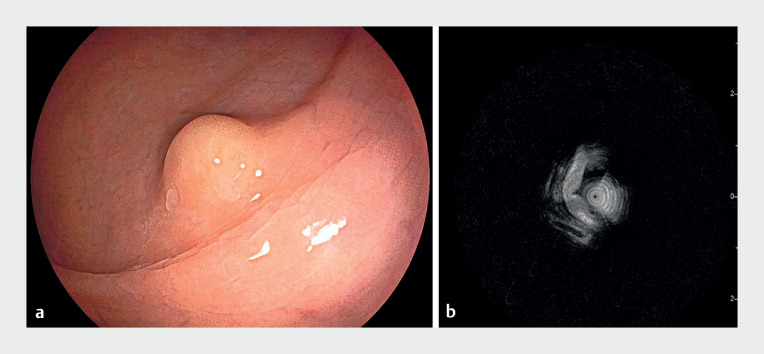
A subepithelial lesion in the rectum, measuring approximately 7 mm in diameter. **a** On endoscopy, the lesion had a yellowish appearance. **b** Endoscopic ultrasound indicated the lesion’s location in the deep mucosal and submucosal layers, adjacent to the muscularis propria.

**Fig. 2:**
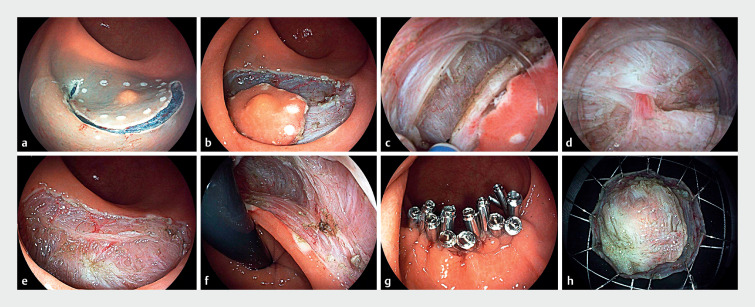
The process of underwater endoscopic intermuscular dissection. **a** Circumferential electrocoagulation was used to mark the edges of the lesion, followed by submucosal injection with normal saline and indigo carmine. **b** Circumferential incision was performed outside the markers, until the muscle layer was exposed. **c** Muscle fibers of the circular part of the muscle layer were cut off to gain access to the intermuscular space under water immersion. **d** Blood vessels were distinctly observable under water immersion. **e,f** Postoperative defect showing longitudinal muscle with no perforations and absence of circular muscle. **g** Closure of the defect with metal clips. **h** The resected tumor exhibited distinctly discernible muscular layers.

The effectiveness of underwater endoscopic intermuscular dissection in the management of rectal neuroendocrine tumors.Video 1

The patient underwent endotracheal intubation under general anesthesia. Marking, submucosal injection, and mucosal incision procedures were performed similarly to endoscopic submucosal dissection (ESD). Following exposure of the muscularis propria, an ST-Hood (DH-33GR; Fujifilm, Tokyo, Japan) was attached to the tip of the endoscope. Subsequently, the circular muscle was incised circumferentially with a 2-mm knife (ORISE ProKnife; Boston Scientific, Marlborough, Massachusetts, USA) under saline solution immersion, thereby unveiling the intermuscular space. Dissection within the intermuscular space was performed until the tumor was completely dissected. Postoperative histopathologic examination revealed a well-differentiated NET (grade 1), with negative horizontal and vertical margins ( Fig. 3 ). The patient was discharged after 72 hours, with no adverse events.

**Fig. 3:**
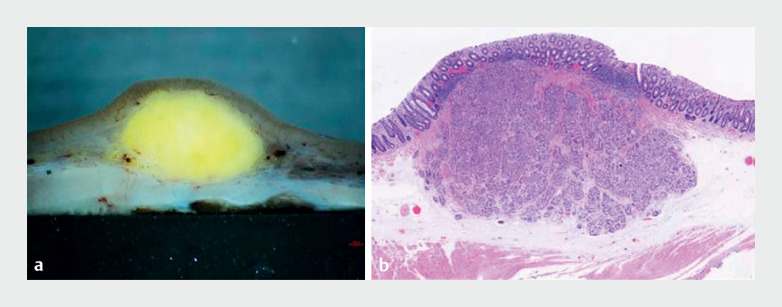
The tumor specimen. **a** The excised tumor after 24 hours of fixation. It presented a yellowish hue, with intrinsic muscular layers visible underneath when viewed from the side. **b** A well-differentiated neuroendocrine tumor (grade 1), with negative horizontal and vertical margins.

Underwater ESD has been documented in the literature 4 5 . Employing water methodologies during EID procedure unveils distinct advantages. The intrinsic buoyancy of water, combined with its magnifying properties, enhances the differentiation of the intermuscular spaces. This heightened clarity facilitates the seamless and expeditious dissection of muscular layers, enabling precise visualization of pulsatile vasculature, thereby facilitating timely interventions to mitigate hemorrhagic complications. The duration of the surgery was 40 minutes. To the best of our knowledge, this case represents the first reported instance of a rectal NET treated with U-EID, thereby substantiating the efficacy and safety of implementing U-EID in the management of rectal diseases.

Endoscopy_UCTN_Code_TTT_1AQ_2AD_3AD

## Notes

Endoscopy E-Videos https://eref.thieme.de/e-videos
*E-Videos* is an open access online section of the journal *Endoscopy* , reporting on interesting cases and new techniques in gastroenterological endoscopy. All papers include a high-quality video and are published with a Creative Commons CC-BY license. Endoscopy E-Videos qualify for HINARI discounts and waivers and eligibility is automatically checked during the submission process. We grant 100% waivers to articles whose corresponding authors are based in Group A countries and 50% waivers to those who are based in Group B countries as classified by Research4Life (see: https://www.research4life.org/access/eligibility/ ). This section has its own submission website at https://mc.manuscriptcentral.com/e-videos .
